# A novel framework of classical and quantum prisoner’s dilemma games on coupled networks

**DOI:** 10.1038/srep23024

**Published:** 2016-03-15

**Authors:** Xinyang Deng, Qi Zhang, Yong Deng, Zhen Wang

**Affiliations:** 1 School of Computer and Information Science, Southwest University, Chongqing, 400715, China; 2 Center for Quantitative Sciences, Vanderbilt University School of Medicine, Nashville, TN, 37232, USA; 3 Big Data Decision Institute, Jinan University, Tianhe, Guangzhou, 510632, China; 4 School of Automation, Northwestern Polytechnical University, Xi’an, 710072, China

## Abstract

Evolutionary games on multilayer networks are attracting growing interest. While among previous studies, the role of quantum games in such a infrastructure is still virgin and may become a fascinating issue across a myriad of research realms. To mimick two kinds of different interactive environments and mechanisms, in this paper a new framework of classical and quantum prisoner’s dilemma games on two-layer coupled networks is considered. Within the proposed model, the impact of coupling factor of networks and entanglement degree in quantum games on the evolutionary process has been studied. Simulation results show that the entanglement has no impact on the evolution of the classical prisoner’s dilemma, while the rise of the coupling factor obviously impedes cooperation in this game, and the evolution of quantum prisoner’s dilemma is greatly impacted by the combined effect of entanglement and coupling.

Cooperation behavior is ubiquitous in natural world and human society. Because of its great significance, understanding the cooperation, for example how cooperation emerges, what promotes cooperation, how cooperation behavior evolves and so on, has become a fundamental issue in evolutionary biology and attracted growing interest for long time^1^,^2^. Evolutionary game theory has offered a theoretical framework to study cooperation behavior among selfish individuals in interactive decision situations^3^,^4^,^5^,^6^,^7^. Among numerous game models, the prisoner’s dilemma (PD) game has attracted the most attention and become a classical and paradigmatic metaphor to study this issue^8^,^9^,^10^,^11^,^12^,^13^,^14^,^15^,^16^. Besides getting concerns from communities of classical game theory, this game model is also studied in the form of quantum version by physicists^17^. However, relative to the classical counterpart, quantum prisoner’s dilemma (QPD) game exhibits some completely different characteristics, one of which is that original dilemma can be resolved via quantum strategies. Though the respective evolution of PD and QPD has attracted much attention in previous studies^18^,^19^,^20^,^21^, the co-evolution of PD and QPD is rarely considered. With this regard, here classical and quantum PD will be coupled together to study the co-evolution of classical games and quantum games.

Up to now, a great number of factors promoting cooperation have been identified. Typical examples includes punishment and reward^22^,^23^,^24^, social diversity^25^, role assignation^26^,^27^, structured population^8^,^28^, age and memory^29^,^30^, heterogeneous activity^31^, to name but a few^32^,^33^,^34^,^35^. Recently, Nowak attribute all these scenarios to five well-known reciprocity mechanisms^36^: kin selection, direct reciprocity, indirect reciprocity, network reciprocity, and group selection. Among them, network reciprocity, also known as spatial reciprocity, has been inspired the most research enthusiasm^37^,^38^,^39^,^40^. Generally, evolutionary games on networks can be classified into two categories based on the located interaction structure: evolutionary games on simplex networks, and games on multilayer networks, respectively. The former mainly include scale-free networks^41^, small world networks^42^, etc. The multilayer networks, as a more flexible and versatile structure, have gained more and more attention recently^43^,^44^,^45^,^46^,^47^,^48^,^49^,^50^,^51^ (see a recent review^52^ for more details).

Among previous studies of coupled network games, game models are almost classical version where the quantum entanglement, representing an underlying connection among individuals, is not taken into consideration. The research paradigms are basically “classical games — classical games”, but not “classical games — quantum games” or “quantum games — quantum games”. In this paper, we turn our attention to the co-evolution of classical and quantum games. The PD, including two strategies cooperation (*C*) and defection (*D*), is used to model the interaction in macro world located on one network. The QPD, including three strategies cooperation (*C*), defection (*D*), and super cooperation (*Q*), is used to model the interaction in quantum world which is also located on another network. Like^24^,^46^, these two networks are coupled by utility functions, and their coupling degree is measured by a coupling factor *α* (0 ≤ *α* ≤ 1). In QPD, an entanglement degree *γ*, where 0 ≤ *γ* ≤ *π*/2, is employed to represent the entanglement. Thus, we mainly study the impact of *α* and *γ* on the co-evolution of PD and QPD on the coupled networks.

## Methods

### Prisoner’s dilemma game and its quantum counterpart

The prisoner’s dilemma (PD) game is a simple and paradigmatic metaphor to study cooperation between unrelated individuals. In its basic version, there are two players who can choose either cooperation (*C*) or defection (*D*), respectively. In accordance with common practice^8^, the temptation of defection *T* = *b* determines the payoff received by a defector when meeting a cooperator, the reward for mutual cooperation is *R* = 1, the punishment for mutual defection is defined by *P* = 0, and the sucker’s payoff *S* = 0 is the payoff received by a cooperator if meeting a defector, where 1 < *b* < 2. It is clear that defection is the unique Nash equilibrium (NE) and evolutionarily stable strategy (ESS) of the PD. The payoff matrix of PD is expressed as


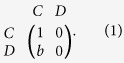


Quantum prisoner’s dilemma (QPD) game is an extension of classical PD by introducing quantum strategies and quantum entanglement. In the seminal work of Eisert *et al.*^17^, a widely used quantization scheme has been developed to extend classical PD into the quantum domain. In Eisert *et al.*’s scheme, the strategy of each player is in the form of unitary operator:


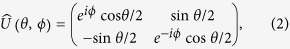


with 0 ≤ *θ* ≤ *π* and 0 ≤ *ϕ* ≤ *π*/2. Specifically, the identity operator 
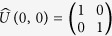
 and bit-flip operator 
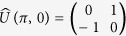
 correspond to the classical “cooperation” and “defection” strategies, respectively. In Eisert *et al.*’s scheme, there is another parameter *γ* which is a measure for the game’s entanglement, 0 ≤ *γ* ≤ *π*/2. For a separable game with *γ* = 0, strategy 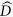, namely 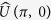, is the Nash Equilibrium (NE), and the QPD does not exhibit any features that go beyond PD. But for a maximally entangled quantum game with *γ* = *π*/2, a new strategy 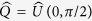 becomes the NE where the *Pareto efficiency* has been realized, in that sense the dilemma in the classical PD is removed.

Eisert *et al.*’s scheme is a little hard to be understood and accepted by the community of classical game theory because it involves some elusive quantum concepts and complicated computing. Li and Yong^18^ has simplified Eisert *et al.*’s scheme to a concise version by reducing the strategic space. In Li and Yong’s version of QPD, it consists of three strategies: cooperation (*C*), defection (*D*), and super cooperation (*Q*), given by 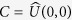, 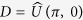, and 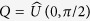, respectively. The payoff matrix is shown as below.


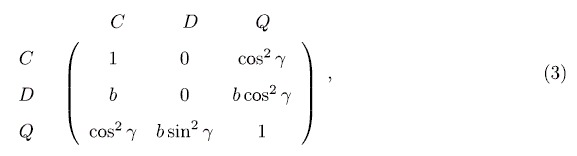


where *γ* is an entanglement degree, 0 ≤ *γ* ≤ *π*/2. In the simplified QPD, although there is only a quantum strategy *Q*, it still displays basic features of the quantum prisoner’s dilemma game: (1) If *γ* = 0, strategy *Q* collapses at *C* and the QPD degenerates to a classical PD; (2) If *γ* = *π*/2, quantum strategy *Q* is the unique NE which has realized Pareto efficiency; (3) If a *Q* meets a *C*, *Q* does not take advantage from *C*, and they get the same payoffs cos^2^*γ*; (4) With the increase of *γ*, the *Q* gets more and more from the game playing against *D*, and gradually becomes a dominant strategy with respect to *D*.

### Evolutionary model of PD and QPD on coupled networks

In this paper, we consider the co-evolution of classical PD and simplified QPD on coupled networks consisting of two-layer scale-free networks. The coupled network is constructed as follows. First, the Barabási-Albert algorithm^53^ is used to produce two identical scale-free networks *G*_1_ and *G*_2_ with size *N*_1_ = *N*_2_ = *N*. Then, we randomly select a node *v*_1_ from *G*_1_ and a node *v*_2_ from *G*_2_ to build an external link between them. Repeat this process for the remainder nodes of networks till each node on *G*_1_ has a corresponding partner on *G*_2_, and the converse is also true. As a result, the coupled structure is constructed by adding *N* external links to *G*_1_ and *G*_2_. The framework of this coupled infrastructure can get many kinds of explanations in the real world. For example, it can be seen as a real contacting network of people and its corresponding online social network. The key feature of the coupled networks is that each node has different connections on different layers. Although a corresponding relation is built between two networks *G*_1_ and *G*_2_, they are not physically connected, but are correlated by the utility function:


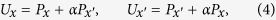


where *x* is a node on *G*_1_ and *x*′ is the coupled node on *G*_2_, and *P*_*x*_ and *P*_*x*′_ are payoffs of *x* and *x*′ received from each network they belong to, respectively. Parameter *α* represents the coupling factor between *G*_1_ and *G*_2_, where 0 ≤ *α* ≤ 1. If *α* = 0, the two layers of coupled networks become completely independent.

In the evolutionary process, classical PD is placed on network *G*_1_, and the QPD is on *G*_2_. The evolutionary process of PD and QPD on the constructed coupled networks is simulated via the Monte Carlo simulation procedure comprising the following elementary steps. Initially, each player on *G*_1_ is randomly designated either as *C* or *D*, and players on *G*_2_ are randomly designated as one role from *C*, *D*, and *Q*. Then, every player from either *G*_1_ or *G*_2_ acquires its accumulated payoff by playing the game with all its linked neighbors on the same network (layer). More specifically, a player on *G*_1_ just plays games with its neighbors on *G*_1_, and the same to players on *G*_2_. Once players on *G*_1_ and *G*_2_ received their payoffs, the utility of each player is computed according to Eq. (4). Next, at each Monte carlo step (MCS) players update their strategies in an asynchronous manner: (i) we randomly select a player *x* from *G*_1_, then player *x* chooses a neighbor *y* on *G*_1_ at random and adopts the strategy *s*_*y*_ from player *y* with a probability


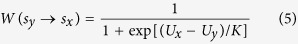


where *K* = 0.1 denotes the uncertainty related to the strategy adoption process and its value does not qualitatively affect the evolutionary outcomes^4^; (ii) Similarly, execute operation (i) on network *G*_2_; (iii) Repeat (i) and (ii) *N* times, where *N* is the size of networks *G*_1_ and *G*_2_, so that each player on both *G*_1_ and *G*_2_ has a chance to change its strategy once on average during this MCS. Then the coupled networks go to next round of evolution.

In this paper, the simulation is performed on two identical scale-free networks *G*_1_ and *G*_2_ with size *N*_1_ = *N*_2_ = *N* = 5000 and average degree 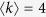. The stationary fraction of each strategy is determined as the average within the last 1000 out of the total 5 × 10^4^ MCS.

## Simulation Results

**Figure 1:**
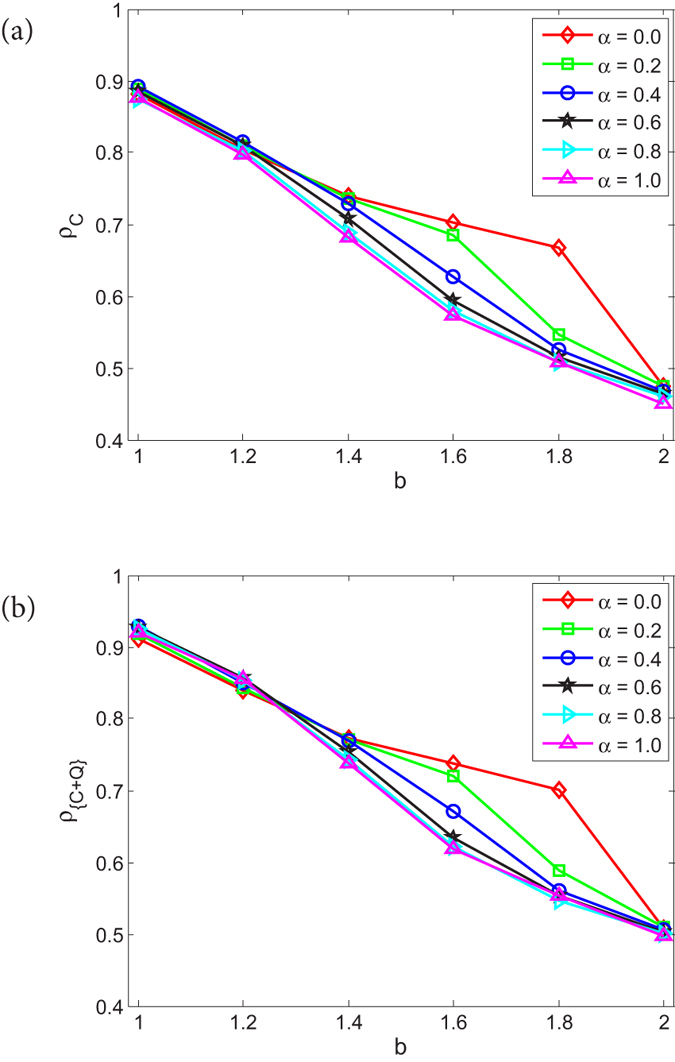
The stationary fraction of different strategies as a function of temptation of defect *b* when *γ* = 0. (**a**) corresponds to the PD on network *G*_1_, and (**b**) is associated with the QPD on network *G*_2_. Each data presented is the average of 100 realizations.

We first investigate a special case of *γ* = 0 in which there dose not exist quantum entanglement so that the QPD degenerates to classical PD, and the evolution on networks *G*_1_ and *G*_2_ is mainly affected by coupling factor *α*. Essentially, this case is equivalent to the evolution of PD on two-layer networks where layers are coupled by parameter *α*. It is easily found that the evolution on each network is completely irrelevant to the other one if *α* = 0. Figure 1 reveals the stationary fraction of different strategies as a function of *b* when *γ* = 0 on the coupled networks. Figure 1(a) corresponds to classical PD on *G*_1_, Fig. 1(b) is the case of QPD on *G*_2_. From Fig. 1(a), for the PD on *G*_1_: (i) despite the coupling factor *α*, the fraction of cooperators *ρ*_*C*_ drops with the increase of *b*; (ii) the smaller the value of *α* is, the better cooperators survive, such that when *α* = 0 representing networks *G*_1_ and *G*_2_ become completely independent, cooperation is promoted at the most extent. For the QPD on *G*_2_, as shown in Fig. 1(b), the obtained results are same with the case of PD on *G*_1_ because strategy *Q* collapses at strategy *C* and the QPD completely degenerates to classical PD when *γ* = 0. In summary, it is found that the coupling between networks *G*_1_ and *G*_2_ is not beneficial for cooperation when the entanglement is not taken into consideration.

**Figure 2:**
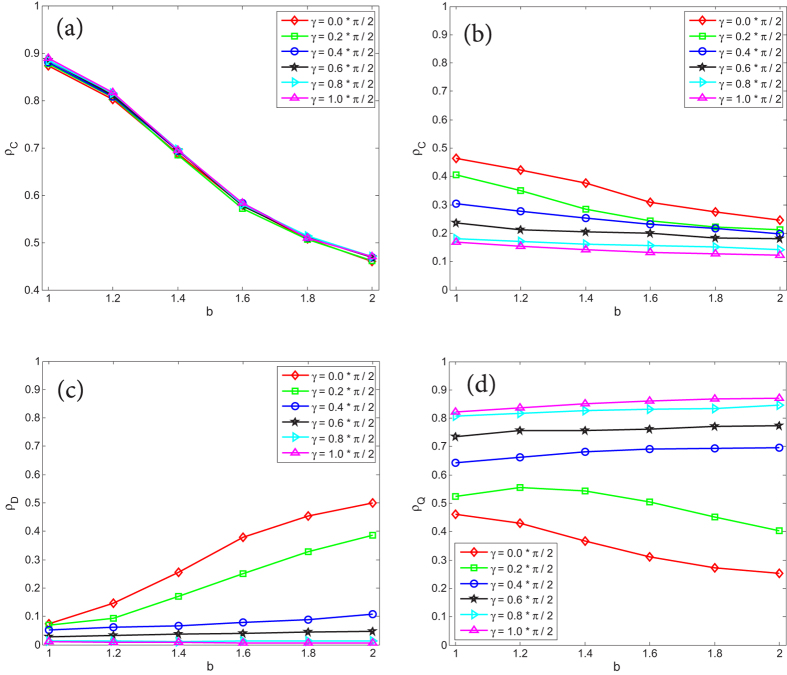
Given *α* = 0.8, the stationary fraction of different strategies as a function of *b* when *γ* takes different values for PD and QPD. (**a**) is the result of *ρ*_*C*_ of PD on network *G*_1_; (**b**–**d**) correspond to *ρ*_*C*_, *ρ*_*D*_, and *ρ*_*Q*_ of QPD on network *G*_2_, respectively. Each data presented is the average of 100 realizations.

Next, to illustrate the impact of entanglement degree *γ* on the evolution of PD and QPD on the coupled networks, we fix the coupling factor *α* = 0.8 so as to observe the stationary fraction of different strategies when *γ* takes different values. For PD on *G*_1_, Fig. 2(a) shows *ρ*_*C*_ as a function of *b* under different *γ*, given *α* = 0.8. Correspondingly, for QPD on *G*_2_, Fig. 2(b–d) present the curves of *ρ*_*C*_, *ρ*_*D*_, *ρ*_*Q*_, respectively. From Fig. 2(a), it is found that the *ρ*_*C*_ curve of PD on *G*_1_ basically does not show any changes regarding *γ*, which means the entanglement degree *γ* of QPD on *G*_2_ does not influence the evolution of PD on *G*_1_.

For QPD on network *G*_2_, there are three kinds of strategies (i.e., cooperator *C*, defector *D*, super cooperator *Q*), from figures their stationary fractions change with *γ*, monotonously: with the rise of *γ*, *ρ*_*C*_ and *ρ*_*D*_ decline (see Fig. 2(b,c)), *ρ*_*Q*_ increases (see Fig. 2(d)). These results indicate that high entanglement degree is beneficial for *Q*, and harmful to *C* and *Q* in the evolution of QPD on network *G*_2_. A theoretical analysis can be made to help understand the results. Let us recall the payoff matrix of QPD, as shown in Eq. (3). Li and Yong’s study^18^ shows that quantum strategy *Q* is the unique NE and ESS when 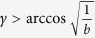. Meanwhile, if the population is well-mixed, with the process of evolution, *C* and *D* are going to be extinct, and *Q* will gradually occupy the whole population. However, instead of a well-mixed population, in this paper the evolution of QPD is on a scale-free network which provides a structured population setting, so that in the end of evolution strategies coexist in the population and the stationary fraction of each strategy is varying with *γ*. In order to better understand what happens, let us decompose the payoff matrix Eq. (3) into pair-wise forms:


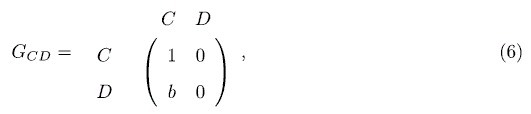



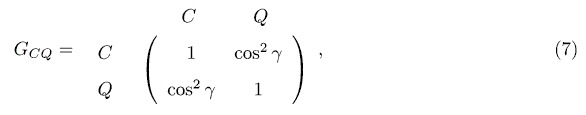



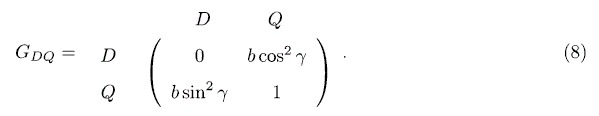


It is found that: (i) in the game *G*_*CD*_, *D* is strictly dominant compared with *C*, but *C* can get a certain amount of payoffs if a cluster of *C*s has formed; (ii) in game *G*_*CQ*_, *C* and *Q* have equal status, anyone can not take advantage from the other, and when *γ* ≠ 0 either *C* or *Q* must to cluster together to get the maximum payoff 1; (iii) in game *G*_*DQ*_, *Q* receives *b* sin^2^*γ* or 1, *D* obtains nothing or *b* cos^2^*γ*, and with the increase of *γ* the expected payoff of *D* gets smaller and smaller until 0. By summarizing these points and noticing the population is located on a scale-free network, we get more understandings about the underlying mechanism of evolutionary QPD on *G*_2_: (i) with the rise of *γ*, *Q* can get more and more by exploiting *D* so that the payoff of *Q* exceeds that of *C*, as a result *ρ*_*Q*_ will be higher than *ρ*_*C*_; (ii) Because the population is located on a scale-free network structure, *C* can form clusters to resist the invasion of *D*, and to contend against *Q*; As *γ* gets bigger and bigger, the disadvantage of *C* becomes more and more obvious so that *ρ*_*C*_ has to go down until reaching the minimum when *γ* = *π*/2; (iii) As *γ* rises, on the one hand *D* gets less and less in the game against *Q*, on the other hand *D* fails to invade clusters of *C*s, so that *ρ*_*D*_ declines and almost becomes 0 while *γ* = *π*/2.

**Figure 3:**
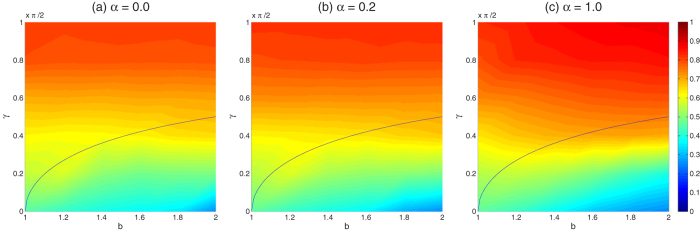
Color map encoding the fraction of strategy *Q* (i.e. *ρ*_*Q*_) on the *b* − *γ* parameter plane. (**a**–**c**) correspond to the cases of *α* = 0.0, *α* = 0.2, and *α* = 1.0, respectively. The curve on each map follows equation 
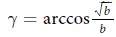
.

In Fig. 2(d), we have shown that strategy *Q* can not totally dominate network *G*_2_, the stationary fraction of *Q*, *ρ*_*Q*_, just reaches a high level (but not 100%) even if the entanglement degree *γ* takes the maximum value *π*/2. This result is a little inconsistent with previous study shown in literature^18^. In Li and Yong’s study^18^, the authors drew a conclusion that super cooperator *Q* will emerge and basically dominates the whole population if 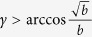, and they verified the conclusion by using experiments evolved on random graphs. However, in our simulation, except of *Q*, at the end of evolution the population always contains a certain quantity of *C*. Someone may argue that in our simulation, shown in Fig. 2, the evolution of QPD occurred in two-layer coupled structures where the coupling factor *α* was set as 0.8, but not in a single-layered network. In order to eliminate the possible dispute, Fig. 3(a) shows the color map encoding *ρ*_*Q*_ on the *b* − *γ* parameter plane when *α* = 0.0. For the sake of comparison, the cases of *α* = 0.2 and *α* = 1.0 are also given in Fig. 3(b,c). Due to *α* = 0.0 in Fig. 3(a), this case is equivalent to the setting of QPD evolved on a single-layered network. From Fig. 3(a), it is found that: (i) on a scale-free network the fraction stationary of strategy *Q* is evidently less than 1 when *γ* is larger than 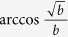, even when *γ* = *π*/2; (ii) if *γ* is obviously larger than 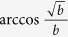, *ρ*_*Q*_ determined by the same *γ* is basically unchanged for any *b*; (iii) if *γ* is obviously smaller than 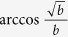, *Q* is gradually suppressed with the rise of the temptation of defect *b*. Therefore, through Fig. 3(a), we have clearly shown that Li and Yong’s conclusion is not fully satisfied on a scale free network. Although strategy *Q* is the unique NE and ESS of QPD when 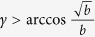, but the feature only guarantees that in that condition *Q* will dominate all nodes of well-mixed populations or some random graphs whose heterogeneity are very low, as shown in Li and Yong’s study^18^. For high heterogeneous populations, such us scale free networks, our study shows that *C* can resist the invasion of evolutionarily stable and NE strategy *Q* so that *C* and *Q* coexist at the end of evolutionary QPD game. The result has provided a complement and perfection to previous study^18^. In addition, from Fig. 3(a–c), we also find that the low sensitivity of *ρ*_*Q*_ to *b* for the same *γ* which is obviously larger than 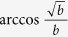 is maintained and *ρ*_*Q*_ becomes bigger as *α* rises. And if *γ* is obviously smaller than 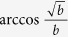, the negative correlation between *ρ*_*Q*_ and *b* is amplified with the increase of *α*.

**Figure 4:**
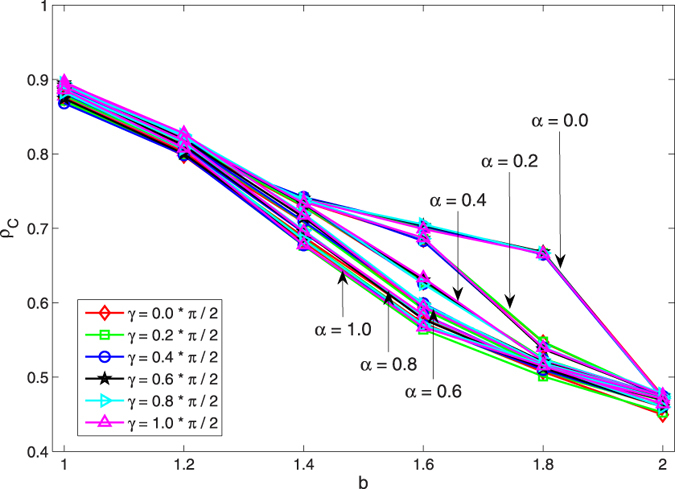
The stationary fraction of cooperators in evolutionary PD on network *G*_1_ as a function of *b* under different *α* and *γ*. Each data presented is the average of 100 realizations.

Then, let us further study how the coupling factor *α* impacts the evolution of various strategies in PD and QPD games on the coupled networks. Similarly, we respectively consider the evolution of PD on *G*_1_ and QPD on *G*_2_. Figure 4 presents the stationary fraction of cooperators in evolutionary PD on network *G*_1_ as a function of temptation of defect *b* under different coupling factor *α* and entanglement degree *γ*. From Fig. 4, two main results are found as follows. Firstly, as same as Fig. 2(a), given a value of *α*, the entanglement degree *γ* basically does not have influences on *ρ*_*C*_ of evolutionary PD on *G*_1_. Secondly, the stationary fraction of cooperators *ρ*_*C*_ is increasing monotonously with the decline of *α*, and it gets the maximum values when *α* = 0. The results show that low coupling factor is better to promote cooperation in classical PD in the coupled strructure.

**Figure 5:**
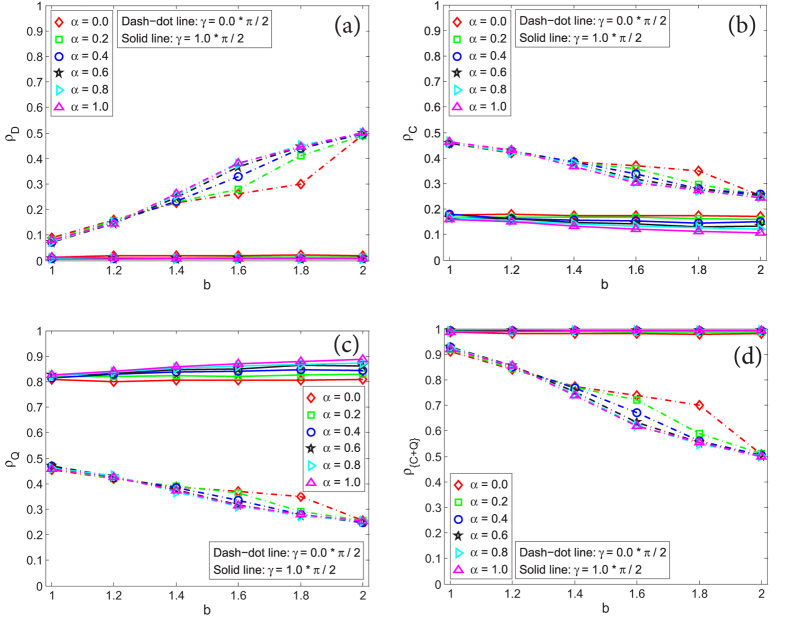
The stationary fraction of strategies as a function of *b* in the evolutionary QPD on network *G*_2_ under different *α* and *γ*. (**a**–**d**) correspond to results of *ρ*_*D*_, *ρ*_*C*_, *ρ*_*Q*_, and *ρ*_{*C*+*Q*}_, respectively. Each data presented is the average of 100 realizations.

For QPD on network *G*_2_, it is a little complicated. Let us investigate the impact of *γ* and *α* on *ρ*_*D*_, *ρ*_*C*_, *ρ*_*Q*_, *ρ*_{*C*+*Q*}_, respectively, as shown in Fig. 5. With respect to *γ*, herein we just consider two extreme cases *γ* = 0 and *γ* = *π*/2 because Fig. 2 has shown that the stationary fraction of each strategy changes monotonously with *γ*. First, from Fig. 5(a), it shows again that in the QPD strategy *D* basically can not survive in the case of maximum entanglement *γ* = *π*/2. And if there is no entanglement (i.e., *γ* = 0 so that the QPD degenerates to classical PD), the maximum coupling between *G*_1_ and *G*_2_ can best elevate the fraction of *D* in the evolutionary QPD on *G*_2_. Second, from Fig. 5(b) we find that the entanglement is not beneficial for *C* generally, and despite of *γ* no coupling (i.e., *α* = 0) is always better to promote strategy *C* in the QPD on network *G*_2_. Moreover, we also notice that the strategy *C* does not go extinct even in the worst situation. Third, let us consider strategy *Q* according to Fig. 5(c). From Fig. 5(c), it is found that the coupling factor has different effects in the cases of maximum-entanglement and no-entanglement: If *γ* = *π*/2 meaning that it has the maximum entanglement in the QPD, the coupling plays a positive effect on boosting *ρ*_*Q*_; However, if *γ* = 0 which means there is no entanglement so that strategy *Q* collapses at *C*, the coupling plays a negative effect on *ρ*_*Q*_. Last, we go to study the sum of *ρ*_*C*_ and *ρ*_*Q*_, namely *ρ*_{*C*+*Q*}_, under different *γ* and *α*, as shown in Fig. 5(d). According to Fig. 5(d), the effect of coupling on *ρ*_{*C*+*Q*}_ becomes very small when *γ* = *π*/2, and at that case the population is basically composed by *C* and *Q*. When *γ* = 0 where *Q* collapses at *C* and the QPD degenerates to the PD, the coupling of *α* = 1 declines *ρ*_{*C*+*Q*}_ to the greatest extent.

**Figure 6:**
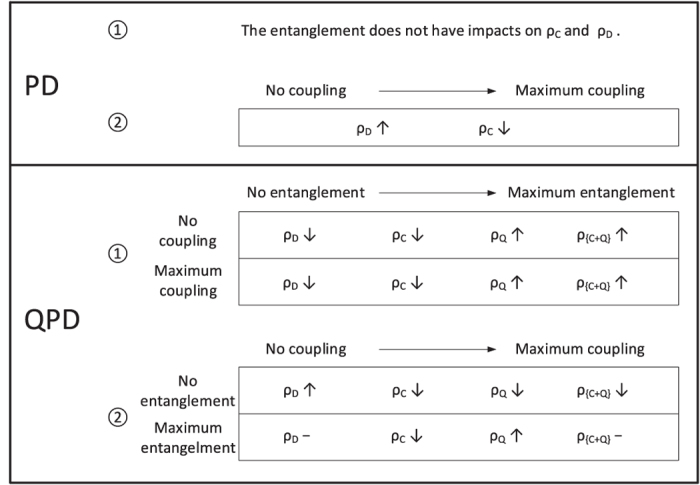
Summary of the evolution of PD and QPD on the two-layer coupled networks. Symbols “↑”, “↓”, and “−” represent ascending, declining, and unchanged, respectively, when the entanglement degree *γ* or coupling factor *α* changes from 0 to maximum.

Now, based on the simulation results mentioned above, we can basically summarize the impacts of the coupling factor *α* and entanglement degree *γ* on the evolution of PD and QPD on two-layer coupling networks, as shown in Fig. 6. For the PD on *G*_1_: (i) the entanglement has no impacts on the evolution of strategies *C* and *D*; and (ii) despite *γ*, with the rise of coupling factor *α* the fraction of cooperators *ρ*_*C*_ is going down. However, for the QPD on *G*_2_: (i) either if *α* = 0 or *α* = 1, as the entanglement degree *γ* increases, strategies *D* and *C* are suppressed so that *ρ*_*D*_ and *ρ*_*C*_ decline, while *ρ*_*Q*_ gets a high increase which counteracts the decline of *ρ*_*C*_ so that *ρ*_{*C*+*Q*}_ can also increase; (ii) if *γ* = 0, the increase of the coupling factor *α* makes *ρ*_*D*_ rising, *ρ*_*C*_ and *ρ*_*Q*_ declining; if *γ* gets the maximum value, the increase of *α* makes *ρ*_*C*_ declining and *ρ*_*Q*_ rising, but basically does not impact *ρ*_*D*_ and *ρ*_{*C*+*Q*}_.

**Figure 7:**
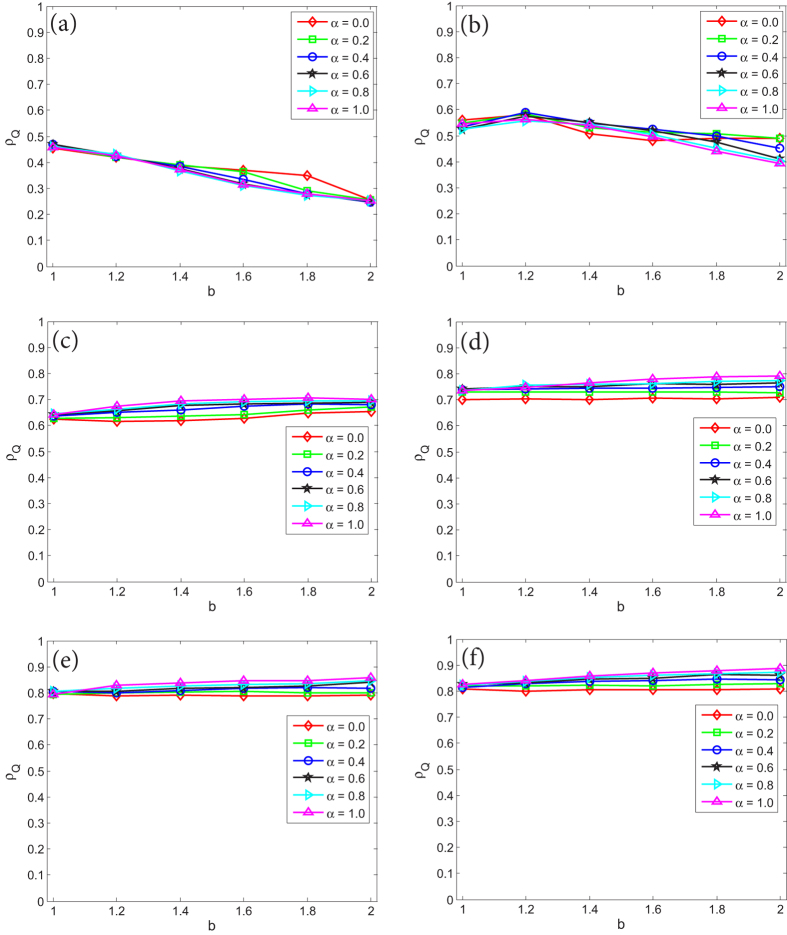
The stationary fraction of super cooperators (*Q*) as a function of *b* in the evolutionary QPD on network *G*_2_ under different *α*, given that the entanglement degree *γ* takes different values. (**a**–**f**) correspond to results of *γ* being 0.0 ∗ *π*/2, 0.2 ∗ *π*/2, 0.4 ∗ *π*/2, 0.6 ∗ *π*/2, 0.8 ∗ *π*/2, 1.0 ∗ *π*/2, respectively. Each data presented is the average of 100 realizations.

In Fig. 5(c), we have simply shown that for the QPD the coupling factor *α* has positive effect on *ρ*_*Q*_ if *γ* = *π*/2, and negative effect on *ρ*_*Q*_ if *γ* = 0. Now let us deeply explore the impact of *α* on *ρ*_*Q*_ when the entanglement degree *γ* takes different values, as shown in Fig. 7. From Fig. 7(a) where *γ* = 0.0 ∗ *π*/2, it is found that: (i) on the one hand, *ρ*_*Q*_ is decreasing with the increase of the temptation of defect *b*; (ii) on the other hand, strategy *Q* is suppressed to the most degree when *α* gets the biggest value 1. However, as *γ* rises, these two points are challenged. If *γ* = 0.2 ∗ *π*/2 (see Fig. 7(b)), although *ρ*_*Q*_ declines barely as *b* increases, the effect of *α* becomes disordered so that the fraction of *Q* is not always the smallest when *α* = 1. Moreover, as shown in Fig. 7(c–e), starting from *γ* = 0.4 ∗ *π*/2, a new pattern is gradually built up that (i) as *b* increases, *ρ*_*Q*_ is either unchanging or boosted; (ii) strategy *Q* is promoted to the most degree when *α* gets the biggest value 1. At the most extreme case where *α* = 1.0 and *γ* = *π*/2, *ρ*_*Q*_ is 0.8270 at *b* = 1.0 and 0.8879 at *b* = 2.0, which shows that the rise of temptation of defect *b* does not restrain strategy *Q*. And at the case with *γ* = *π*/2 and *b* = 2.0, *ρ*_*Q*_ becomes 0.8095 at *α* = 0.0 and 0.8879 at *α* = 1.0, which means the increase of coupling factor *α* does not impede the super cooperator *Q*. These cases reflect that quantum strategy *Q* has many fantastic features which are obvious different from classical cooperation strategy *Q* that is always suppressed by the increase of *b* or *α* on single-layered or two-layer networks.

**Figure 8:**
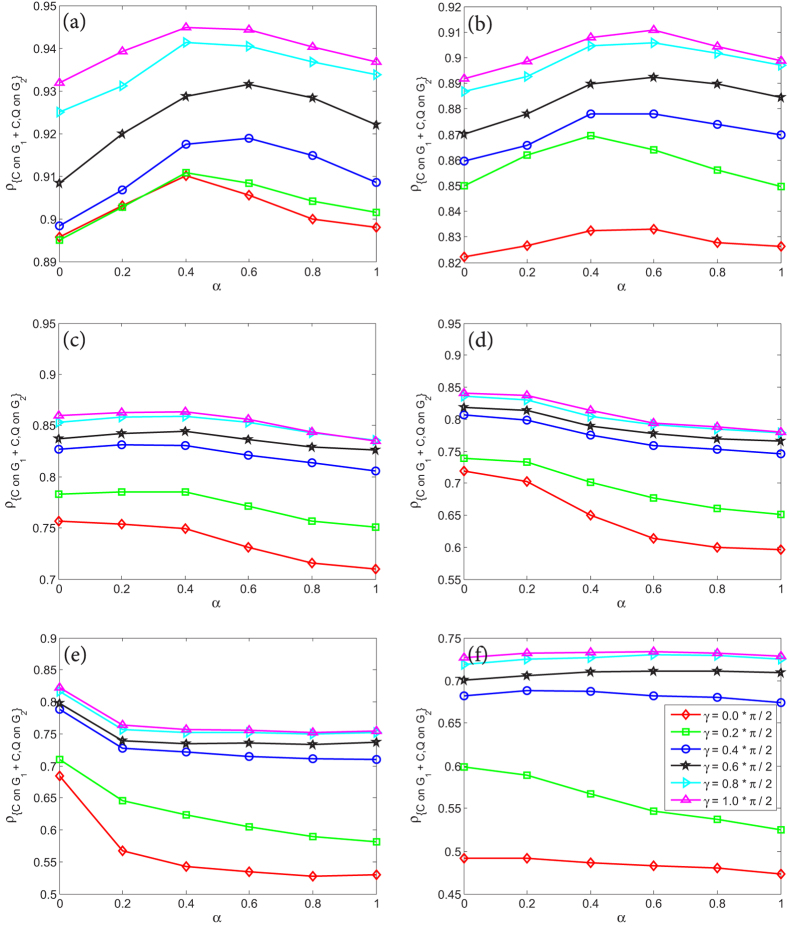
The stationary fraction of all cooperators, including *C* on *G*_1_ and *C*, *Q* on *G*_2_, on the whole two-layer coupled network as a function of *α* under different *γ*, given that the temptation of defect *b* takes different values. (**a**–**f**) correspond to results of *b* being 1.0, 1.2, 1.4, 1.6, 1.8, 2.0, respectively. Each data presented is the average of 100 realizations.

Lastly, let us investigate how to increase the fraction of cooperators including *C* and *Q* on the whole two-layer coupled networks. Figure 8 shows the stationary fraction of all cooperators, including *C* on *G*_1_ and *C*, *Q* on *G*_2_, on the coupled networks as a function of *α* under different *γ*, given that the temptation of defect *b* takes different values. It is found that: (i) In any cases, the entanglement is forever beneficial to boosting the fraction of cooperators on the whole two-layer coupled networks. The bigger the value of *γ*, the more the cooperators on the coupled networks. (ii) If *b* gets a small value, such as 1.0 or 1.2 (see Fig. 8(a,b)), for different entanglement degree *γ*, the peak of the stationary fraction of all cooperators is located at an intermediate point of *α* belonging to [0, 1] but not at the smallest point of *α*, although at the peak point the fraction of cooperators is just slightly higher than that of others. (iii) If *b* gets a bigger value, for example 1.4 or 1.6 or 1.8 (see Fig. 8(c–e)), regardless of *γ*, the quantity of all cooperators will reach the maximum value when coupling factor *α* = 0.0. And in most cases the promotion for the fraction of cooperators at *α* = 0.0 is relatively obvious, in comparison of that with *α* = 1.0. (iiii) If *b* gets the maximum value 2.0 (see Fig. 8(f)), the reduction of *α* can not remarkably enhance cooperation when entanglement degree *γ* is high. Inspired by these observations mentioned above, in order to promote the cooperation level of the whole coupled structure as much as possible, therefore, we can adopt the following strategy: At first, if *b* is very large, for example *b* = 2.0, we’d better invest the most effort to increase the entanglement degree *γ* of QPD, which can most efficiently enhance the cooperation level of whole coupled networks; Second, if *b* is a little big, such as 1.4 < *b* < 1.8, we must simultaneously increase the entanglement degree *γ* and reduce the coupling factor *α* as much as possible; Third, if *b* is small, such as 1.0 < *b* < 1.2, in order to elevate the cooperation level of the whole network, we should, one the one hand enhance the entanglement degree *γ*, one the other hand find an appropriate value of the coupling factor *α* but not just reduce it.

## Conclusion

In this paper we have shown the co-evolution of classical PD and simplified QPD on two-layer coupled networks. Different from previous studies of evolutionary games on multi-layer networks, in this work classical games and quantum games are coupled together. Classical prisoner’s dilemma and quantum prisoner’s dilemma are employed as the models of two different interactive environments and mechanisms, respectively. And two pivotal parameters, the entanglement degree *γ* in the quantum game and coupling factor *α* between classical games and quantum games, are taken into consideration. By means of numerous simulations, the main results in the evolution process are concluded basically. We hope this study can provide new insight in the evolutionary games on multilayer networks and understanding for the social dilemmas.

## Additional Information

**How to cite this article**: Deng, X. *et al.* A novel framework of classical and quantum prisoner's dilemma games on coupled networks. *Sci. Rep.* **6**, 23024; doi: 10.1038/srep23024 (2016).
